# Exploring Neighborhood Influences on Small-Area Variations in Intimate Partner Violence Risk: A Bayesian Random-Effects Modeling Approach

**DOI:** 10.3390/ijerph110100866

**Published:** 2014-01-09

**Authors:** Enrique Gracia, Antonio López-Quílez, Miriam Marco, Silvia Lladosa, Marisol Lila

**Affiliations:** 1 Department of Social Psychology, University of Valencia, Valencia 46010, Spain; E-Mails: mimar4@alumni.uv.es (M.M.); marisol.lila@uv.es (M.L.); 2 Department of Statistics and Operations Research, University of Valencia, Burjassot 46100, Spain; E-Mails: antonio.lopez@uv.es (A.L.-Q.); silvia.lladosa@uv.es (S.L.)

**Keywords:** Bayesian spatial modeling, crime, disorder, immigration, intimate partner violence, neighborhoods, social environment, social disorganization

## Abstract

This paper uses spatial data of cases of intimate partner violence against women (IPVAW) to examine neighborhood-level influences on small-area variations in IPVAW risk in a police district of the city of Valencia (Spain). To analyze area variations in IPVAW risk and its association with neighborhood-level explanatory variables we use a Bayesian spatial random-effects modeling approach, as well as disease mapping methods to represent risk probabilities in each area. Analyses show that IPVAW cases are more likely in areas of high immigrant concentration, high public disorder and crime, and high physical disorder. Results also show a spatial component indicating remaining variability attributable to spatially structured random effects. Bayesian spatial modeling offers a new perspective to identify IPVAW high and low risk areas, and provides a new avenue for the design of better-informed prevention and intervention strategies.

## 1. Introduction

The serious physical, mental, and social consequences of intimate partner violence against women (IPVAW), and its high prevalence worldwide, make it a major social and public health problem [1,2,3,4,5,6,7]. Recently, the World Health Organization published a report considering violence against women as a “global public health problem of epidemic proportions, requiring urgent action” ([7], p. 3). According to this report, the global lifetime prevalence of intimate partner violence among ever-partnered women is 30% (95% CI = 27.8% to 32.2%) and 23.2% in the high-income regions (95% CI = 20.2% to 26.2%). IPVAW is a complex phenomenon whose understanding needs to go beyond individual factors to include the wider social environment [2,8,9,10,11]. Although research has traditionally focused more on personal and situational factors, scholars are increasingly stressing the importance of a more ecological approach to understanding and preventing IPVAW, and acknowledging the influence of community and neighborhood-level variables, both as IPVAW risk and protective factors.

It has been long recognized the link between neighborhood-level characteristics and rates of violence (among non-intimates) in communities. This research tradition, drawing mainly from social disorganization theories, posits that characteristics of neighborhoods such as disadvantage, poverty, ethnic heterogeneity, residential instability, disorder, or diminished collective efficacy undermine social control and facilitates crime and violence [12,13,14,15,16,17,18]. This ecological approach emphasizing neighborhood-level influences on violence was also appealing to scholars studying violence in intimate relationships, mainly child abuse [19,20,21,22,23]. More recently, the influence of neighborhood-level variables on IPVAW is also receiving increased scholarly attention [24,25,26,27,28,29,30].

A growing body of research, also drawing mainly from social disorganization theories, is examining the influence of a number of neighborhood structural characteristics (e.g., concentrated disadvantage, ethnic heterogeneity, residential instability) and processes (e.g., collective efficacy, neighborhood social ties, cultural norms) on the incidence of IPVAW [30,31]. Among these characteristics, neighborhood concentrated disadvantage (measured in a variety of ways such as poverty, unemployment, or social and physical disorder) appears to be the more consistent predictor of rates of IPVAW [32,33,34,35,36,37,38], even when risk factors at the individual level are controlled for [32,33]. Lack of resources, poor socioeconomic conditions, and high levels of exposure to disorder and violence in these communities may explain this link as they increase levels of stress among residents, foster a culture of tolerant attitudes towards violence (both in general and in particular among intimates), and reduce levels of social control, which in turn facilitates the incidence of IPVAW [31,33,39,40,41,42,43,44,45]. Evidence regarding the influence of social processes linked to social disorganization theories is still very limited, but available evidence suggests that whereas social ties and support between neighbors and collective efficacy are protective factors for IPVAW, violence-accepting neighborhood norms may increase rates of IPVAW in those neighborhoods [30,31,38]. Evidence regarding relationships between other social structural characteristics of neighborhoods such as ethnic heterogeneity or residential instability on the IPVAW incidence is mixed and less conclusive [30].

This body of research underscores the idea that beyond individual-level risk factors, macrosocial or neighborhood-level variables such as concentrated disadvantage may play an important role in explaining rates of IPVAW. Accordingly, an unequal distribution of neighborhood-level risk factors would result in an unequal distribution of IPVAW risk within these areas. As neighborhood-level risk factors are clustered in space and are unequally distributed within communities, and there may be other unobserved spatially structured influences on risk, a spatial perspective appears to be particularly appropriate in order to analyze variations in IPVAW risk across neighborhoods accounting for spatial dependence. However, despite the growing interest in neighborhood-level variables influencing IPVAW, research using spatial analytical methods to examine IPVAW risk variations across neighborhoods, except for few exceptions, is almost non-existent [37]. Clearly, a more detailed understanding of this relationship from a spatial modeling approach may contribute to better-informed intervention and prevention strategies addressing this major social and public health problem.

Although Bayesian spatial modeling is common in disease mapping [46,47,48,49], its use in the field of crime and violence studies is still quite uncommon. A number of scholars, however, are increasingly recognizing the advantages of Bayesian spatial modeling to study crime and violence (among non-intimates) as compared to past research using non-spatial analytical methods or other non-Bayesian spatial methods, and a small but growing number of studies are beginning to use this approach [50,51,52,53,54]. However, its application to the study of IPVAW is still very rare [37]. Addressing this gap in the literature, this paper aims to contribute to a better understanding of neighborhood-level influences on IPVAW by analyzing small-area variations in IPVAW risk. Bayesian hierarchical models are a very useful tool for incorporating geographical information into the regression analysis of small-area data. They allow the mapping of spatial components which express the trend of geographical variation. In addition, these models are able to deal with uncertainty in a sequential way through prior distributions on parameters and hyperparameters. A Bayesian random-effects modeling approach will be used to address issues of spatial autocorrelation and overdispersion that may arise when using small-area count data, and which allows to take into account other unmeasured spatially structured influences on risk [50,51,52,53,54,55,56]. We aim to test whether a set of neighborhood characteristics, meaningful in terms of the social disorganization theory (*i.e.*, area socioeconomic status, percentage of immigrant population, police reported levels of public disorder and crime, and observed social and physical disorder), as well as other unobserved spatially clustered influences, can explain small-area variations in IPVAW risk.

## 2. Data

This research was conducted in Valencia, the third largest city in Spain, with a population of 797,028. The Valencia Police Department divides the city in seven police districts, and for this study we used data from the 5th police district. This district covers a city area with a population of 237,320. We used data from this police district as this was the only one in the city with a long period of IPVAW records with geographical data, which provided more stable estimates of partner IPVAW cases. Based on police information, it can be assumed that this police district is representative of the city as a whole and that it also resembles other cities in the range of IPVAW across neighborhoods (*i.e.*, this is not a particular high or low IPVAW section of the city). For analysis we used the minimum administrative unit available: *census block groups* [57,58,59]. Census block groups are usually smaller than census tract units, and generally are defined by “walkable” areas of a little number of city blocks that make them particularly adequate to study neighborhood influences [57]. In this study census block groups contained approximately between 800 and 2,600 residents. The selected police district consisted of 80 census block groups, with an average population of 1,476 residents.

*Protection Orders.* For the purpose of this study we use IPVAW cases with associated protection orders. These cases represent the serious end of IPVAW cases, described in the literature as what has been called “intimate terrorism” as opposed to common couple violence [60,61]. According to 2012 official data, IPVAW cases with associated protection orders represent 16.53% of all reported cases. The use of serious cases of IPVAW make this study unique as compared to other studies looking at the link between partner violence and neighborhood characteristics, where data is usually based on other sources such as police calls or anonymous surveys [24,33,37].

The IPVAW data for this study provided by the Valencia Police Department consists of all protection orders dictated between 1 January 2007 and 31 March 2013 (*N* = 368). All cases of partner violence were male to female violence, as all protections orders were for IPVAW. The *protection order* is dictated by a court of law after evaluating the severity of the offense and recognizing the existence of an objective risk for the victim. This order is enforced by trained police officers that are available for any arising emergency or other IPVAW issues. These orders provide the victim with a comprehensive protection statute that includes police protection with the aim of imposing physical distance between the aggressor and the victim as immediate protection from further violence, as well as civil (e.g., shelters) or penal (e.g., restraining order) actions, along with social assistance measures. It is important to note that in Spain, as opposed to other countries, a protection order is an exceptional measure used for serious cases of IPVAW. Removal of these protection orders is also decided by the court of law (*i.e.*, victims cannot drop these protection orders when they wish).

**Figure 1:**
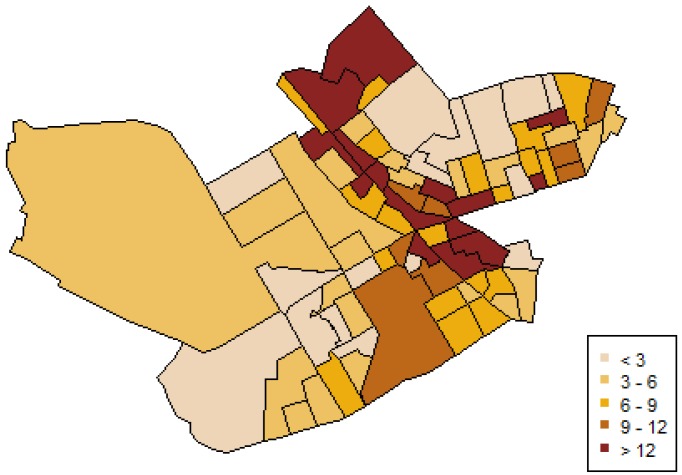
Incidence rates of intimate partner violence against women (IPVAW).

For geocoding IPVAW data the geographic coordinates of the street address where the incident motivating the protection order happened were obtained [62]. To perform spatial analyses, counts of protection orders in each census block group were summed and weighted by the population of women 16 years-old and over, creating an intimate partner violence rate. The census block groups range from 0 to 16 cases of intimate partner violence, with an average of 4–5 cases by census block group, and rates from 0 to 30.37 per thousand people in the census block groups (see Figure 1).

*Census block group data.* The Statistics Office of Valencia City Hall provided socioeconomic data for each census block group corresponding to the year 2011. We used as an indicator of neighborhood socioeconomic status the average *cadastral property value* (*i.e.*, an administrative value of a property set by the local Town Hall Authority as a reference for fiscal and for other administrative purposes such as public subsidies). As an indicator of ethnic heterogeneity we used the *immigrant population percentage*. Finally, as an indicator of residential instability we used an index of *residential mobility* (*i.e.*, the proportion of population moving in and moving out, per 1,000 inhabitants) (see Table 1).

**Table 1:** Variables (mean, standard deviation, minimum and maximum values) at the census block group level.

Variable	Mean	SD	Min.	Max.
Population	1,476	439.61	829	2,559
Women > 16 years	651.3	191.15	361	1,174
Property value	22,440	9,160	11,190	52,580
% Immigration	16.58	6.85	6.33	33.17
Policing activity	10.16	3.56	2	18
Social Disorder	0.29	0.75	0	4
Physical Disorder	6.2	3.26	0	16
Residential Mobility	22.92	6.01	11.39	34.52

*Disorder.* Neighborhood disorder refers to observed or perceived physical and social features of neighborhoods that may signal the breakdown of order and social control, and that can undermine the quality of community life [63,64,65,66]. Two trained raters walked each census block group in order to complete a 20-item scale that evaluates neighborhood disorder. It is a Likert-type scale with a 5-point response (0 = *No presence*, 4 = *Highly present*). The scale is composed by two factors: Physical disorder (e.g., cigarrete butts and litter in the street, graffiti, vacant or abandoned housing, vandalized and run-down buildings), and Social disorder (e.g., people loitering, people drunk or taking drugs on the streets, fights, drug-dealing or street prostitution). Raters were not allowed to discuss a particular rating as they conducted a census block group. Disorder data collection was limited to 16:00 to 21:00 hours [67].

*Policing activity.* Public disorder and crime was obtained using an index of policing activity provided by the staff of the police district. This index included interventions in violent and drug related crimes, fights, public disorder, vandalism, social incivilities, public drunkenness, homeless people, truancy, *etc.* The policing activity index ranges from 0 (*very low*) to 4 (*very high*).

## 3. Methods

Since the dependent variable is a count outcome (number of protection orders for IPVAW), it is assumed to follow a Poisson distribution (a strictly positive and discrete distribution). More specifically, if *O_i_* represents counts of protection orders for IPVAW in each of the *i* census block groups, we assumed that *O_i_* ~ Possion(*λ_i_E_i_*), where *E_i_* is the expected number of protection orders for IPVAW and *λ_i_* is the area specific risk in location *i*.

A first model, specifically a Poisson regression model, was assessed including six explanatory variables: property value, immigration rates, social and physical disorder, policing activity and residential instability. The log(*E_i_*) was included in the model as an offset term to control size differences of areal units.

To explore the linkages between the effects of explanatory variables described above and the spatial autocorrelation as well as the overdispersion, we used a Poisson hierarchical regression model specified in Equation (1) as:

log(*λ_i_*) = α + *X_i_*β + *S_i_* + *H_i_*, *i* = 1, …, *n* (1)

where α is the total mean (intercept), β represents the vector of the regression coefficients, *X_i_* is the matrix of covariates in the census block group *i* (*i* = 1, …, *n*) and *S* and *H* are two random effects terms to account for spatial autocorrelation and overdispersion respectively.

The spatially correlated heterogeneity component *S* has been specified by a conditional spatial autoregressive (CAR) model, which relates the expected value at each location with the observations in adjacent locations. It is defined as follows:

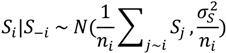
(2)
where *n_i_* is the number of neighborhoods of census block group *i*, *S_-i_* indicates the values of *S* vector except the *i* th component, the expression *j~i* denotes all units *j* neighborhoods of area *i* and *σ_S_* is the standard deviation parameter.

Following a Bayesian approach, the parameters are treated as random variables and therefore prior knowledge is incorporated via prior distributions. Specifically, we use vague Gaussian distributions *N* (0, 100000) for the fixed effects β and an improper uniform distribution for α. The random unstructured heterogeneity (*H*) is specified as a normal distribution *N* (0, 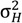) where *σ_H_* ~ *U* (0,1). The prior information of standard deviation of spatial effect is also a uniform distribution *σ_S_* ~ *U* (0,1).

Bayesian estimation is carried out using the software R and the WinBUGS package, generating with Markov Chain Monte Carlo (MCMC) multiple samples of the parameters of the statistical model. We generated a total of 100,000 iterations discarding the first 10,000 iterations as a burn-in period of MCMC. To check the convergence of the simulated sequences we used the convergence diagnostic 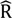 [68] which was near to 1.0 for all parameters. A sensitivity analysis on prior distributions of hyperparameters was performed to measure the robustness of the results. The posterior distributions showed the consistency of results. Finally, models were compared by considering the Deviance Information Criterion (DIC) [69], which is computed routinely by WinBUGS. The model with the smaller DIC value was chosen.

## 4. Results

The final model selected using the DIC criterion included the percentage of immigrant population, physical disorder and policing activity as covariates, a spatial component that accounted for the spatial autocorrelation, and an unstructured random effect to control for Poisson overdispersion.

Results of Bayesian regression models are presented in Table 2, showing the posterior mean and the 95% credible interval (CI) of both fixed (β) and random effects (*σ_S_* and *σ_H_*), as well as the DIC value and the effective number of parameters (*p_D_*). This table summarizes the results of three models. Model 1 is a non-spatial Poisson regression. This Model only includes the six covariates described above. In this model, the property value and residential instability was negative associated with IPVAW, whereas the rest of covariates showed a positive association. Before carrying out a selection of relevant variables the model was re-fitted (Model 2). Model 2 included the spatial and heterogeneity components, and improved the DIC value. In Models 1 and 2 all regression coefficients for the six covariates had the same sign.

In contrast to frequentist methodology, the Bayesian credible intervals are interpreted in probability terms. The posterior distribution of parameters shows the probability of a negative or positive association, and allows assessing their relevance of these. In this regard, property value, social disorder (DS) and residential instability did not have a clear association with IPVAW. These covariates were regarded as non-relevant and, consequently were removed from Model 2. The results are shown in Table 2 (Model 3).

**Table 2:** Results of non-spatial and spatial Poisson regression from WinBUGS.

Explanatory Variables	Non-spatial Poisson (Model 1)	Spatial Poisson (Model 2)	Final Spatial Model (Model 3)
	Mean (95% CI)	Mean (95% CI)	Mean (95% CI)
Intercept	−1.154 (−1.965, −2.99)	−1.221 (−2.291, −0.153)	−1.715 (−2.193, −1.253)
Property Value ^a^	−0.104 (−0.331, 0.102)	−0.092 (−0.359, 0.175)	--
Immigration	0.046 (0.011, 0.080)	0.049 (0.006, 0.095)	0.046 (0.026, 0.064)
Policing Activity	0.056 (0.022, 0.093)	0.057 (0.016, 0.099)	0.064 (0.0287, 0.104)
Social Disorder	0.025 (−0.102, 0.148)	0.036 (−0.134, 0.199)	--
Physical Disorder	0.034 (0, 0.07)	0.030 (−0.013, 0.074)	0.030 (−0.009, 0.071)
Residential Instability	−0.010 (−0.042, 0.001)	−0.012 (−0.052, 0.029)	--
σ*_S_*	--	0.232 (0.012, 0.587)	0.232 (0.010, 0.576)
σ*_H_*	--	0.205 (0.015, 0.407)	0.190 (0.004, 0.378)
DIC	355.6	353.7	348.9
*p_D_*	6.9	25.1	21.193

Note: ^a^ This variable was included as the cadastral value divided by 1,000 to solve computational problems with the prior distributions assigned to fixed effects.

**Figure 2:**
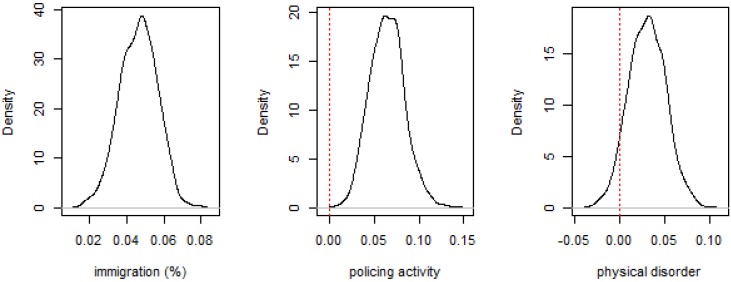
Posterior distribution of fixed effect in the final model (Model 3).

In the final spatial model (Model 3), immigrant percentage, policing activity and physical disorder had a strong positive association with the outcome variable (*i.e.*, the posterior probability of being different from zero was greater). Figure 2 shows the posterior distributions of fixed effects. This indicates that the number of cases of IPVAW is greater in areas with higher percentage of immigrants, higher levels of policing activity and higher physical disorder. When the models were fitted using the same prior distribution for all regression parameters, the DIC for Model 3 was 348.9, as compared to 355.6 for Model 2. This indicates that Model 3 fits the data better than Model 2.

One advantage of the spatial methodology is the ability to illustrate maps that allows visualizing areas of high risk of IPVAW, as well as the effect of the spatial component.

**Figure 3:**
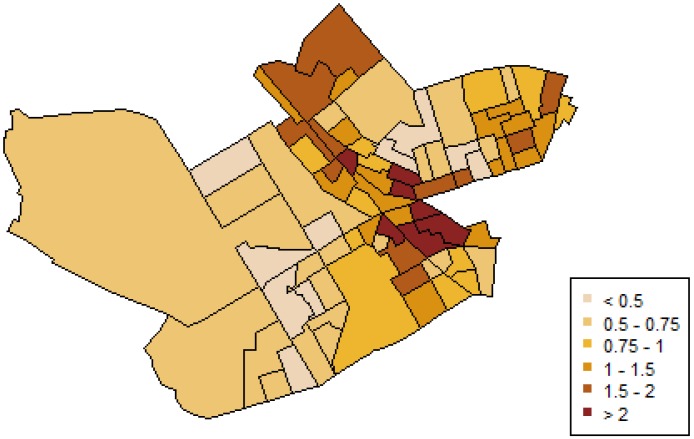
Risk map.

We only report maps from the final spatial model. Figure 3 represents the posterior mean risk *λ_i_* of IPVAW in each census block group. The mapped risk includes the effects of the covariates, the spatial autocorrelation and the overdispersion according to Equation (1). Each individual value represents the relative risk compared to the whole district incidence. Figure 3 shows that risks greater than one cover most of the eastern zone, with some areas where the risk exceeds twice the average value.

**Figure 4:**
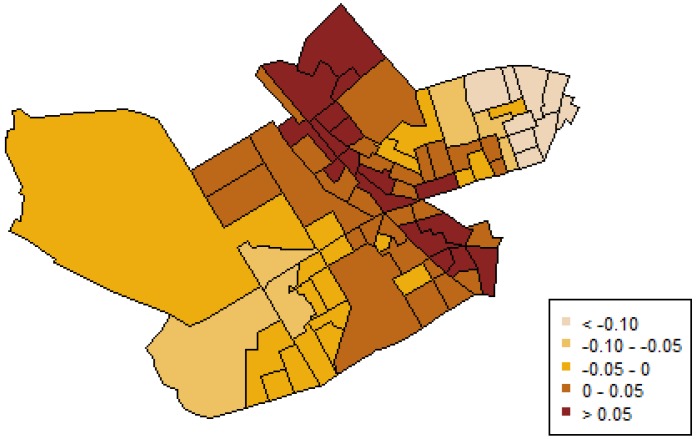
Posterior mean of the spatial component of IPVAW incidence.

Beyond the fixed effects of the covariates, the geographical variation of IPVAW incidents is modeled by the spatially structured random effect. This spatial component (Figure 4) showed a strong effect, with positive values in the center of the region. It represents the geographical pattern that could not be explained by the explanatory covariates in the model. These maps provide information about locations with high risk, where more attention is needed.

## 5. Discussion and Conclusions

In this study we used spatial data of IPVAW cases to examine neighborhood-level influences on small-area variations of IPVAW risk in a police district of the city of Valencia (Spain). Drawing from past research on neighborhood influences on IPVAW, we have explored the influence of six neighborhood structural characteristics, meaningful in terms of social disorganization theories, on the spatial distribution of IPVAW cases: area socioeconomic status (measured in terms of the property value), police reported levels of public disorder and crime, observed physical and social disorder, ethnic heterogeneity (measured in terms of percentage of immigrant population), and residential instability (measured in terms of residential mobility). To analyze area variations in IPVAW risk and its association with neighborhood-level explanatory variables we used a Bayesian spatial random-effects modeling approach, as well as disease mapping methods to represent risk probabilities in each area. This study represents a significant contribution to the extant literature as this spatial epidemiological perspective is still seldom used in crime analysis [50,51,52,53,54], and almost non-existent in studies on IPVAW [37].

Our analyses showed the relevance of three of the predictors examined in explaining the spatial distribution of IPVAW cases, and also revealed a spatial component indicating remaining variability attributable to spatially structured random effects. These analyses indicated that cases of IPVAW are more likely in areas with high immigrant concentration, high levels of public disorder and crime, and high levels of physical disorder.

The positive association between immigrant concentration and IPVAW is particularly interesting, as the available literature on the influence of ethnic heterogeneity on rates of IPVAW is contradictory [30]. Previous studies have found either no effect of neighborhood-level immigrant concentration on IPVAW, or even a negative association with the incidence of IPVAW [38]. This negative relationship, found particularly in areas with high concentration of Latino immigrants (thus named as the “Latino paradox” or immigrant paradox), suggests a protective effect of high immigrant concentration on levels of IPVAW [70]. The reason why it has been suggested that immigrant concentration has a buffering effect on IPVAW is that it brings unique social ties and cultural norms. However, low sociocultural status, acculturation stress, the loss of social ties and the challenges associated to the immigrant status, as well as different gender roles attitudes (more accepting and tolerant of partner violence) have been also considered in the literature as important factors explaining the increased risk for IPVAW among minority and immigrant groups [71]. In Spain higher rates of IPVAW are found consistently among the immigrant population, in particular Latin-American immigrants [71]. In this regard, the clear association between immigrant concentration and IPVAW incidence found in the present study is more in line with other studies showing higher rates of IPVAW among immigrant women [72,73,74], and those suggesting that higher levels of IPVAW among immigrants are mediated in part by the community context where they live—highly disadvantaged environments characterized by poverty, segregation, and social isolation [75]. Our results are also in line with research showing a contextual effect of concentrated immigration on other types of violence [76,77].

Our results also showed that cases of IPVAW are more likely in areas with high levels of policing activity (motivated by public disorder and crime), as well as in areas characterized by high physical disorder. Findings regarding the link between areas with high public disorder and criminal activity (including violence) that attracts high levels of police activity, connects with previous research linking perceptions and exposure to neighborhood violence to IPVAW incidence rates [41,43,78,79,80]. Observed social disorder, although in the expected direction (a positive association with IPVAW incidence), was considered not significant in our final model. However, high levels of policing activity are also an indicator of social disorder, and taken together with physical disorder, a variable traditionally linked to street-level violence [14,66], our findings suggest that disorder is also a significant predictor of higher rates of IPVAW.

These findings provide evidence that neighborhood characteristics are important influences not just on street-level violence (among non-intimates) as social disorganization theories posit, but that this influence also extends to violence among intimates occurring “behind closed doors”. In this sense this study adds to the debate on why neighborhood contextual effects on street-level crime and violence also influence violence among intimates that takes place in the privacy of the home [14,15,17,70]. As to why this neighborhood effects also operate inside the home, thus affecting IPVAW rates, the debate remains open, although a number of possible explanations have been put forward [28,30,33,41,70]. According to social disorganization theorizing, disorder and crime may increase the sense of fear, mistrust and insecurity among residents, diminishing their capacity for collective action and informal control [81]. In this regard, disorder and crime have been considered as outcomes that can be partly explained by neighborhood processes such as collective efficacy [14,15,63]. If we extend this argument to our subject matter, the contextual effect of high levels of disorder and crime on IPVAW rates may also be the result of diminishing informal social control and collective efficacy [28,31]. From this viewpoint, it has been suggested that in neighborhoods with low collective efficacy and weak social ties among neighbors, women victims are more isolated and afraid of disclosing the violence. In these neighborhood residents either do not feel responsible or even if they disapprove IPVAW may be unwilling to help or intervene in “private business” for fear of retaliation, and offenders may feel safer as they do not expect neighbors to intervene [33]. We cannot, however, provide direct evidence for these links, as we do not measure neighborhood processes such as informal social control or collective efficacy. Also, in this regard, evidence on the influence of collective efficacy on IPVAW is still not conclusive, and clearly further research is needed [38].

With respect to the other covariates explored in this study, we used as a proxy to measure neighborhood economic deprivation the property cadastral value. Somewhat surprisingly, this covariate, although in the expected direction (the lower the property value the higher the presence of IPVAW cases), did not reach the criteria to be included in our final model. The link between economic disadvantage and IPVAW is generally supported in the literature [30], which suggests that perhaps our measure of the neighborhood economic status may have not be an adequate measure to tap economic deprivation. Results regarding residential mobility were not meaningful, and are in line with other available research providing mixed evidence on the effects of residential instability on IPVAW [30].

Finally, and regarding areas with higher probabilities of IPVAW, this study showed that variations in IPVAW risk are explained only partly by our three significant covariates, as our results also revealed that there are other spatially structured influences of risk (see Figure 4), which are not accounted for the measured neighborhood-level covariates in the model. This unmeasured spatial component could suggest that other explanatory variables, meaningful in terms of social disorganization theorizing, were not taken into account (e.g., collective efficacy, social ties), or that other processes with spatial structure may be at work. For example, some scholars have considered that shared attitudes of tolerance and acceptance of IPVAW may shape a subculture of legitimization of this type of violence [31,33,41,82,83]. A theoretical possibility is that this subculture could have a spatial correlate corresponding to what Sampson and Lauritsen called “cognitive landscapes or ecologically structured norms (normative ecologies) regarding appropriate standards and expectations of conduct” ([76], p. 63). One would expect higher rates of IPVAW in contexts where violence is not condemned but rather accepted or tolerated to some extent. Clearly these are issues that deserve further research attention.

As for the practical implications of our study, the use of Bayesian spatial modeling for the ecological analysis of small-area variations in IPVAW risk provides a new perspective to better understand the risk factors associated with the spatial distribution of this important social and public health problem. This approach allows the identification of IPVAW high and low risk areas and therefore provides a new avenue for the design of better-informed prevention and intervention strategies. As Congdon noted, “it is important for public health priority setting to identify areas with excess risk and also spatial clustering of excess risk, as evidence of either pattern may provide support for targeted interventions” ([84], p. 5023). Our results suggest that the prevention approach would need to take into account features of the neighborhood context that are unknown in our model. This clearly indicates the need to look more closely at these high risk areas to better understand other variables explaining these levels of risk so they can be identify and used for a better targeted prevention and intervention efforts.

This study has also limitations regarding the covariates used in this study, the generalization of our results, and the setting of the study. As mention above, the measure we use to tap neighborhood economic disadvantaged (*i.e.*, property value) may have not been powerful enough to detect this construct as other variables commonly used in this type of studies such as family income, percentage of people living below the poverty level or unemployment (these measures were not available in the city statistics department). Also, some potentially relevant variables, in terms of social disorganization theory, such as informal social control, collective efficacy, family disruption or social ties were not included in the study as no individual-level data was collected. With respect to the generalization of our results, the IPVAW cases used in this study corresponds to the severe end of violence in intimate relationships, what has been called “intimate terrorism” [61,62], and the spatial distribution and covariates associated to other types of partner violence such as “common couple violence”, or cases drawn from other sources such as police calls or anonymous surveys [24,33,37]. Also in this study all cases of partner violence were male to female violence, as all protections orders were for IPVAW, so results cannot be generalized to female to male partner violence. Another limitation is that we cannot rule out the potential problem of neighborhood selection bias (*i.e.*, intimate partners that choose these high risk neighborhoods, may be already predisposed to IPVAW) [85,86]. Finally, this study was limited to a particular area of the city and our analysis treat the police district examined as a closed system, therefore ignoring potential effects of neighboring areas.

## References

[1] Campbell J.C. (2002). Health consequences of intimate partner violence. Lancet.

[2] World Health Organization (2002). World Report on Violence and Health.

[3] Campbell J., Jones A.S., Dienemann J., Kub J., Schollenberger J., O’campo P., Gielen A.C., Wynne C. (2002). Intimate partner violence and physical health consequences. Arch. Intern. Med..

[4] Coker A.L., Davis K.E., Arias I., Desai S., Sanderson M., Brandt H.M., Smith P.H. (2002). Physical and mental health effects of intimate partner violence for men and women. Am. J. Prev. Med..

[5] Ellsberg M., Jansen H.A., Heise L., Watts C.H., Garcia-Moreno C. (2008). Intimate partner violence and women’s physical and mental health in the WHO multi-country study on women’s health and domestic violence: An observational study. Lancet.

[6] Sarkar N.N. (2008). The impact of intimate partner violence on women’s reproductive health and pregnancy outcome. J. Obstet. Gynecol..

[7] World Health Organization (2013). Global and Regional Estimates of Violence against Women: Prevalence and Health Effects of Intimate Partner Violence and Non-Partner Sexual Violence.

[8] Heise L.L. (1998). Violence against women: An integrated, ecological framework. Violence Against Women.

[9] Van Wyk J.A., Benson M.L., Fox G.L., DeMaris A. (2003). Detangling individual-, partner-, and community-level correlates of partner violence. Crime Delinq..

[10] O’Campo P., Gielen A.C., Faden R.R., Xue X., Kass N., Wang M.C. (1995). Violence by male partners against women during the childbearing year: A contextual analysis. Am. J. Public Health.

[11] Gracia E., Herrero J. (2006). Acceptability of domestic violence against women in the European Union: A multilevel analysis. J. Epidemiol. Community Health.

[12] Shaw C.R., McKay H.D. (1942). Juvenile Delinquency and Urban Areas.

[13] Bursik R.J., Grasmick H.G. (1993). Neighborhood and Crime.

[14] Sampson R.J., Raudenbush S.W., Earls F. (1997). Neighborhood and violent crime: A multilevel study of collective efficacy. Science.

[15] Morenoff J.D., Sampson R.J., Raudenbush S.W. (2001). Neighborhood inequality, collective efficacy, and the spatial dynamics of urban violence. Criminology.

[16] McNulty T.L., Bellair P.E. (2003). Explaining racial and ethnic differences in adolescent violence: Structural disadvantage, family well-being, and social capital. Justice Q..

[17] Browning C.R., Byron R.A., Calder C.A., Krivo L.J., Kwan M.-P., Lee J.-Y., Peterson R.D. (2010). Commercial density, residential concentration, and crime: Land use patterns and violence in neighborhood context. J. Res. Crime Delinq..

[18] Maimon D., Browning C.R. (2010). Unstructured socializing, collective efficacy, and violence behavior among urban youth. Criminology.

[19] Garbarino J., Sherman D. (1980). High-risk neighbourhoods and high-risk families: The human ecology of child maltreatment. Child Dev..

[20] Coulton C.J., Korbin J.E., Su M. (1999). Neighborhoods and child maltreatment: A multilevel study. Child Abuse Negl..

[21] Korbin J.E. (2003). Neighborhood and community connectedness in child maltreatment research. Child Abuse Negl..

[22] Gracia E., Herrero J. (2006). Perceived neighborhood social disorder and residents’ attitudes toward reporting child physical abuse. Child Abuse Negl..

[23] Coulton C.J., Crampton D.S., Irwin M., Spilsbury J.C., Korbin J.E. (2007). How neighborhoods influence child maltreatment: A review of the literature and alternative pathways. Child Abuse Negl..

[24] Miles-Doan R., Kelly S. (1997). Geographic concentration of violence between intimate partners. Public Health Rep..

[25] O’Campo P., Burke J., Peak G.L., McDonnell K.A., Gielen A.C. (2005). Uncovering neighbourhood influences on intimate partner violence using concept mapping. J. Epidemiol. Community Health.

[26] Burke J.G., O’Campo P., Peak G.L. (2006). Neighborhood influences and intimate partner violence: Does geographic setting matter?. J. Urban Health.

[27] Frye V. (2007). The informal social control of intimate partner violence against women: Exploring personal attitudes and perceived neighborhood social cohesion. J. Community Psychol..

[28] Gracia E., Herrero J. (2007). Perceived neighborhood social disorder and attitudes towards reporting domestic violence against women. J. Interpers. Violence.

[29] Frye V., O’Campo P. (2011). Neighborhood effects and intimate partner and sexual violence: Latest results. J. Urban Health.

[30] Pinchevsky G.M., Wright E.M. (2012). The impact of neighborhoods on intimate partner violence and victimization. Trauma Violence Abuse.

[31] Browning C.R. (2002). The span of collective efficacy: Extending social disorganization theory to partner violence. J. Marriage Fam..

[32] Cunradi C.B., Caetano R., Clark C., Schafer J. (2000). Neighborhood poverty as a predictor of intimate partner violence among white, black, and hispanic couples in the United States: A multilevel analysis. Ann. Epidemiol..

[33] Benson M.L., Fox G.L., DeMaris A., Van Wyk J. (2003). Neighborhood disadvantage, individual economic distress and violence against women in intimate relationships. J. Quant. Criminol..

[34] Pearlman D.N., Zierler S., Gjelsvik A., Verhoek-Oftedahl W. (2003). Neighborhood environment, racial position, and risk of police-reported domestic violence: a contextual analysis. Public Health Rep..

[35] Cunradi C.B. (2009). Intimate partner violence among Hispanic men and women: The role of drinking, neighborhood disorder, and acculturation-related factors. Violence Vict..

[36] Li Q., Kirby R.S., Sigler R.T., Hwang S., LaGory M.E., Goldenberg R.L. (2010). A multilevel analysis of individual, household, and neighborhood correlates of intimate partner violence among low-income pregnant women in Jefferson County, Alabama. Am. J. Public Health.

[37] Cunradi C.B., Mair C., Ponicki W., Remer L. (2011). Alcohol outlets, neighborhood characteristics, and intimate partner violence: Ecological analysis of a California city. J. Urban Health.

[38] Wright E.M., Benson M.L. (2011). Clarifying the effects of neighborhood disadvantage and collective efficacy on violence “behind closed doors”. Justice Q..

[39] Jewkes R. (2002). Intimate partner violence: Causes and prevention. Lancet.

[40] Gracia E., Herrero J. (2006). Public attitudes toward reporting partner violence against women and reporting behavior. J. Marriage Fam..

[41] Raghavan C., Mennerich A., Sexton E., James S.E. (2006). Community violence and its direct, indirect, and mediating effects on intimate partner violence. Violence Against Women.

[42] Gracia E., García F., Lila M. (2009). Public responses to intimate partner violence against women: The influence of perceived severity and personal responsibility. Span. J. Psychol..

[43] Reed E., Silverman J.G., Welles S.L., Santana M.C., Missmer S.A., Raj A. (2009). Associations between neighborhood violence and intimate partner violence perpetration among urban, African American men. J. Community Health.

[44] Caetano R., Ramisetty-Mikler S., Harris T.R. (2010). Neighborhood characteristics as predictors of male to female and female to male partner violence. J. Interpers. Violence.

[45] Lila M., Gracia E., Murgui S. (2013). Psychological adjustment and victim-blaming among intimate partner violence offenders: The role of social support and stressful life events. Eur. J. Psychol. Appl. Legal Context.

[46] Clayton D.G., Kaldor J. (1987). Empirical Bayes estimates of age-standardized relative risks for use in disease mapping. Biometrics.

[47] Bernardinelli L., Clayton D., Pascutto C., Montomoli C., Ghislandi M., Songini M. (1995). Bayesian analysis of space-time variation in disease risk. Stat. Med..

[48] Waller L.A., Gotway C.A. (2004). Applied Spatial Statistics for Public Health Data.

[49] Lawson A.B. (2009). Bayesian Disease Mapping: Hierarchical Modeling in Spatial Epidemiology.

[50] Zhu L., Gorman D.M., Horel S. (2006). Hierarchical Bayesian spatial models for alcohol availability, drug “hot spots” and violent crime. Int. J. Health Geogr..

[51] Matthews S.A., Yang T.C., Hayslett K.L., Ruback R.B. (2010). Built environment and property crime in Seattle, 1998–2000: A Bayesian analysis. Environ. Plan. A..

[52] Sparks C.S. (2011). Violent crime in San Antonio, Texas: An application of spatial epidemiological methods. Spat. Spatiotemporal Epidemiol..

[53] Law J., Quick M. (2013). Exploring links between juvenile offenders and social disorganization at a large map scale: A Bayesian spatial modeling approach. J. Geogr. Syst..

[54] Law J., Quick M., Chan P. (2013). Bayesian spatio-temporal modeling for analysing local patterns of crime over time at the small-area level. J. Quant. Criminol..

[55] Congdon P. (2013). Assessing the impact of socioeconomic variables on small area variations in suicide outcomes in England. Int. J. Environ. Res. Public Health.

[56] Jonker M.F., Congdon P.D., van Lenthe F.J., Donkers B., Burdorf A., Mackenbach J.P. (2013). Small-area health comparisons using health-adjusted life expectancies: A Bayesian random-effects approach. Health Place.

[57] The Effects of Using Census Block Groups Instead of Using Census Tracts when Examining Residential Housing Patterns Website. http://www.census.gov/hhes/www/housing/resseg/pdf/unit_of_analysis.pdf.

[58] Sampson R.J., Raudenbush S.W. (2004). Seeing disorder: Neighborhood stigma and the social construction of “broken windows”. Soc. Psychol. Q..

[59] Yonas M., Akers A.Y., Burke J.G., Chang J.C., Thomas A.L., O’Campo P. (2011). Perceptions of prominent neighborhood individuals regarding neighborhood factors and intimate partner violence. J. Urban Health.

[60] Johnson M.P. (1995). Patriarchal terrorism and common couple violence: Two forms of violence against women. J. Marriage Fam..

[61] Johnson M.P., Leone J.M. (2005). The differential effects of intimate terrorism and situational couple violence findings from the national violence against women survey. J. Fam. Issues.

[62] Ratcliffe J.H. (2004). Geocoding crime and a first estimate of a minimum acceptable hit rate. Int. J. Geogr. Inf. Sci..

[63] Sampson R.J., Raudenbush S.W. (1999). Systematic social observation of public spaces: A new look at disorder in urban neighborhoods. Am. J. Sociol..

[64] Skogan W. (1990). Disorder and Decline: Crime and the Spiral of Decay in American Cities.

[65] Taylor R.B. (2001). Breaking away from Broken Windows: Baltimore Neighborhoods and the Nationwide Fight against Crime, Grime, Fear, and Decline.

[66] Wilson J.Q., Kelling G. (1982). The police and neighborhood safety: Broken windows. Atl. Mon..

[67] Perkins D.D., Taylor R.B. (1996). Ecological assessments of community disorder: Their relationship to fear of crime and theoretical implications. Am. J. Community Psychol..

[68] Gelman A., Carlin J., Stern H., Rubin D. (1990). Bayesian Data Analysis.

[69] Spiegelhalter D.J., Best N.G., Carlin B.P., Van der Linde A. (2002). Bayesian measures of complexity and fit. J. R. Stat. Soc..

[70] Wright E.M., Benson M.L. (2010). Immigration and intimate partner violence: Exploring the immigrant paradox. Soc. Probl..

[71] Gracia E., Herrero J., Lila M., Fuente A. (2009). Perception of social disorder in the neighborhood and attitudes toward partner violence against women in Latin-American immigrants. Eur. J. Psychol. Appl. Legal Context.

[72] Raj A., Silverman J.G. (2002). Violence against immigrant women: The roles of culture, context, and legal immigrant status on intimate partner violence. Violence Against Women.

[73] Hazen A.L., Soriano F.I. (2007). Experiences with intimate partner violence among Latina women. Violence Against Women.

[74] Morash M., Bui H., Zhang Y., Holtfreter K. (2007). Risk factors for abusive relationships: A study of Vietnamese American immigrant women. Violence Against Women.

[75] Benson M., Wooldredge J., Thistlethwaite A., Fox G. (2004). The correlation between race and domestic violence is confounded with community context. Soc. Probl..

[76] Sampson R.J., Lauritsen J.L., Reiss A.J., Roth J. (1993). Violent Victimization and Offending: Individual-, Situational-, and Community-Level Risk Factors. Understanding and Preventing Violence: Social Influences.

[77] Sampson R.J., Morenoff J.D., Raudenbush S. (2005). Social anatomy of racial and ethnic disparities in violence. Am. J. Public Health.

[78] Miles-Doan R. (1998). Violence between spouses and intimates: Does neighborhood context matter?. Soc. Forces.

[79] DeKeseredy W.S., Schwartz M.D., Alvi S., Tomaszewski E.A. (2003). Perceived collective efficacy and women’s victimization in public housing. Criminol. Crim. Justice.

[80] Koenig M.A., Stephenson R., Ahmed S., Jejeebhoy S.J., Campbell J. (2006). Individual and contextual determinants of domestic violence in North India. Am. J. Public Health.

[81] Skogan W.G., Reiss A.J., Tonry M. (1986). Fear of Crime and Neighborhood Change. Communities and Crime.

[82] Cunradi C.B. (2010). Neighborhoods, alcohol outlets and intimate partner violence: Addressing research gaps in explanatory mechanisms. Int. J. Environ. Res. Public Health.

[83] Gracia E., Tomás J.M. Correlates of victim-blaming attitudes regarding partner violence against women among the Spanish general population. Violence Against Women.

[84] Congdon P. (2013). Spatially interpolated disease prevalence estimation using collateral indicators of morbidity and ecological risk. Int. J. Environ. Res. Public Health.

[85] Merlo J. (2011). Contextual influences on the individual life course: Building a research framework for social epidemiology. Psychosoc. Interv..

[86] Sampson R.J., Morenoff J.D., Gannon-Rowley T. (2002). Assessing “neighborhood effects”: Social processes and new directions in research. Annu. Rev. Sociol..

